# Changes in Soil Organic Carbon Fractions and Fungal Communities, Subsequent to Different Management Practices in Moso Bamboo Plantations

**DOI:** 10.3390/jof8060640

**Published:** 2022-06-16

**Authors:** Xiaoping Zhang, Qiaoling Li, Zheke Zhong, Zhiyuan Huang, Fangyuan Bian, Chuanbao Yang, Xing Wen

**Affiliations:** 1 China National Bamboo Research Center, Key Laboratory of Bamboo Forest Ecology and Resource Utilization of National Forestry and Grassland Administration, Hangzhou 310012, China; xiaopingzhang@caf.ac.cn (X.Z.); liqiaoling65@163.com (Q.L.); zhiyuanhuang@caf.ac.cn (Z.H.); bianfangyuan@caf.ac.cn (F.B.); chuanbaoy@126.com (C.Y.); wenxing202202@163.com (X.W.); 2 National Long-Term Observation and Research Station for Forest Ecosystem in Hangzhou-Jiaxing-Huzhou Plain, Hangzhou 310012, China; 3 Engineering Research Center of Biochar of Zhejiang Province, Hangzhou 310021, China

**Keywords:** fungi, bamboo, carbon sequestration, carbon management index, ecosystem stability

## Abstract

Moso bamboo (*Phyllostachys pubescens*) has an extremely fast growth rate and major carbon sequestration potential. However, little information is available on the dynamics of soil C accumulation and fungi communities related to different management practices. Here, we investigated changes in the soil organic carbon (SOC) fractions and fungal communities of a Moso bamboo plantation under three different management practices (M0: undisturbed; M1: extensively managed; and M2: intensively managed). Compared with M0, SOC levels were reduced by 41.2% and 71.5% in M1 and M2, respectively; furthermore, four SOC fractions (C1: very labile; C2: labile; C3: less labile; and C4: nonlabile) and the carbon management index (CMI) were also significantly reduced by plantation management. These practices further altered fungal communities, for example, by increasing *Basidiomycota* and *Mortierellomycota*, and by decreasing *Ascomycota* and *Rozellomycota*. *Pyrenochaeta*, *Mortierella*, *Saitozyma*, and *Cladophialophora* were identified as keystone taxa. Soil fungal communities were significantly related to the pH, NH_4_-N, AP, C3, and the C4 fractions of SOC. Random forest modeling identified soil C3 and *Mortierella* as the most important predictors of the CMI. Our results suggest that reducing human interference would be beneficial for fungal community improvement and C sequestration in Moso bamboo plantations.

## 1. Introduction

Bamboo is an important forest resource in subtropical and tropical regions [1] and accounts for 0.80% of the global forest area [2]. Approximately 641.16 million hectares of bamboo forests exist in China, 72.96% of which consist of Moso bamboo [3]. Moso bamboo usually achieves its growth (10–20 m height) and diameter (breast height 8–16 cm) within 35–40 days after its shoots emerge from the soil in the spring [4,5]. Because of its special growth characteristics, this bamboo species is an important non-wood forest product in China [6] and has major carbon (C) sequestration potential [7,8]. Yen and Lee [8] found that Moso bamboo forests have a higher C sequestration rate (8.13 ± 2.15 Mg ha^−1^ year^−1^) than Chinese fir forests (3.35 ± 2.02 Mg ha^−1^ year^−1^). To obtain higher economic benefits, management practices have been implemented in the bamboo plantations [9,10].

Soil represents the largest terrestrial C pool, offering higher C storage than the sum of atmospheric and vegetation C storage [11]. Small changes in the soil C pool have a substantial impact on the global C balance [12]. Forest ecosystems account for more than half of the soil organic carbon (SOC) in terrestrial ecosystems [13,14], and are regarded as the most promising reservoirs for storing additional C and reducing the CO_2_ load [15]. SOC pools comprise labile and non-labile C pools [16,17]. Of these, the labile C pool is subject to rapid turnover rates that influence nutrient cycling, soil quality, and productivity [18]. Conversely, the non-labile C pool is slowly altered by soil microbes [19] and is important for C sequestration [20]. As part of the labile C pool, oxidizable organic carbon (OC) could be an indicator of early dynamic changes to SOC [20,21], because it is more sensitive to changes in the physicochemical properties of soil than total organic carbon (TOC) [22].

Soil fungi are primarily involved in the decomposition of plant material, especially the lignocellulosic components, which are relatively recalcitrant to bacteria. Typically, a succession of fungal species occurs on new substrates when they enter soil, based on the initial decomposition of simpler compounds, followed by the subsequent degradation of more complex polymers [23]. Fungi play a crucial role in SOC dynamics [24,25]. For example, they produce oxidative enzymes that decompose the recalcitrant biopolymers of plant litter [25,26]. Clemmensen et al. [27] found that fungal community shifts might affect soil C decomposition and sequestration, which are closely related to other soil parameters.

Previous studies have shown that management practices can affect SOC dynamics in Moso bamboo plantations [10,28,29]. However, to our knowledge, few studies have been conducted on the relationships between oxidizable OC fractions and soil fungal communities. In this study, we compared the effects of different management strategies on the SOC fractions and fungal communities in Moso bamboo plantations. Our specific objectives were as follows: (1) to determine the effects of different plantation management strategies on SOC fractions and, consequently, on soil C sequestration ability; (2) to evaluate the shifts in the fungal communities of Moso bamboo under different plantation management strategies and the relation thereof to SOC fractions and other soil factors.

## 2. Materials and Methods

### 2.1. Experimental Site and Sample Collection

The field trial was conducted in Tianhuangping town, Anji County, Zhejiang Province, China. The meteorological data of this study area were shown in Yang et al. [10]. The experimental area has a subtropical monsoon climate, with an annual average temperature and sunshine of 17 °C and 1943 h, respectively. This region has 224–240 frost-free days, at altitudes of 300–380 m. The soil is classified as a Ferralsols [30].

Moso bamboo plantations subjected to the following three different management strategies were selected for evaluation: unmanaged (M0), extensively managed (M1), and intensively managed (M2). More information about these bamboo plantations was described by Yang et al. [10]. Briefly, the M0 plantation had not experienced any human management for over 60 years, and gradually developed into a mixed forest. Conversely, certain practices—including the selective and regular harvesting of bamboo trunks and shoots, every two years—were maintained in the M1 plantation, whereas the M2 plantation was subjected to annual selective harvesting, understory vegetation removal, and fertilization, during mid– late June.

In May 2021, we established three comparable stands (M0, M1, and M2) with similar initial site conditions for sample collection. Six 20 × 20 m plots were demarcated in each selected bamboo stand. Within each plot, five S-shaped soil profiles [31] were collected and carefully mixed to form a composite sample for that plot, after being passed through a 2 mm sieve. The fresh soil samples were then divided into two parts, one of which was stored at −80 °C for microbial analysis. The other was air-dried for physicochemical property analysis.

### 2.2. Soil Chemical Analysis

Soil pH was recorded using a glass electrode at a 1:2.5 soil:water (*w*/*v*) ratio. SOC was determined using a TOC analyzer (Multi N/C 3100; Analytik Jena, Jena, Germany). The soil total nitrogen (TN, analyzed using the Kjeldahl method), available phosphorus (AP, extracted using 0.5 M NaHCO_3_), and available potassium (AK, extracted using 1 M ammonium acetate acid) levels were determined, as described by Lu [32]. The concentrations of soil NO_3_-N and NH_4_-N were measured using the indophenol blue colorimetric method [32] and UV spectrophotometry [33], respectively, after extraction at a ratio of 1:5 (soil: 2 M KCl). Because of their degree of oxidizability [34], SOC fractions (C1: very labile; C2: labile; C3: less labile; and C4: nonlabile) were determined using 333, 167, and 33 mmol L^−^^1^ KMnO_4_, respectively, and divided into four constituents.

### 2.3. Calculation of Associations among Parameters

The carbon management index (CMI) was calculated using the following method, described by Blair et al. [35]:

CMI = carbon pool index (CPI) × lability index (LI) × 100(1)

CPI = SOC in soil sample/SOC in M0 soil sample(2)

Lability (L) = labile carbon content/non-labile carbon content(3)

LI = L in soil sample/L in M0 soil sample(4)

### 2.4. Internal Transcribed Spacer (ITS), Gene Sample Preparation, Sequencing, and Analysis

Microbial DNA was extracted using the Fast DNA^®^ Spin Kit for soil (MP Biomedicals, Santa Ana, CA, USA). The ITS1 region was amplified using the primers of ITS1F (5′-CTTGGTCATTTAGAGGAAGTAA-3′) and ITS2 as a reverse primer (5′-GCTGCGTTCTTCATCGATGC-3′). The PCR amplification, library construction, and Illumina MiSeq sequencing were performed by Shanghai Majorbio Bio-pharm Technology Co., Ltd. (Shanghai, China), following the standard protocols [36,37].

### 2.5. Processing of the Sequencing Data

The raw FASTQ files were demultiplexed, quality-filtered using fastp version 0.20.0 [38], and merged using FLASH [39], according to the criteria described by Liu et al. [37]. Operational taxonomic units (OTUs) were clustered using UPARSE [40] at 97% similarity, and chimeric sequences were identified and removed using UCHIME [41]. The taxonomic information of representative OTU sequences were determined using the UNITE (version 8.0; https://unite.ut.ee/, accessed on 1 July 2019) [42] database with a bootstrap cut-off of 80% [43].

### 2.6. Statistical Analyses

All statistical analyses were conducted in R (v4.1.0; http://www.r-project.org/, accessed on 18 May 2021). One-way analysis of variance (ANOVA), followed by least significant difference (LSD) were performed to calculate the difference among the three sites in soil properties, microbial alpha diversity indices, and relative abundances of fungal-dominated phyla and genera. Rarefaction techniques provide a way to adjust the differences in library sizes across samples and aid comparative analysis of alpha diversity [44]. All samples were rarefied to the lowest number of sequences (30,807). Calculation of alpha diversity indices (Shannon and Chao1) and hierarchical clustering were performed in the ‘Vegan’ [45] package. A circos plot was drawn using the ‘circlize’ [46] package, and the network was generated and visualized using ‘ggClusterNet’ [47] package. Only robust correlations (Spearman’s correlation coefficient > 0.6 and *p*-value < 0.05) were in the network. The nodes with high degree in the network were identified as keystone species [48]. Redundancy analysis (RDA) was carried out in the ‘Vegan’ [45] package, to assess the effects of soil factors on the fungal communities. The soil parameters used in the RDA analysis were preselected by the ‘bioenv’ function, and significant soil parameters were determined using the ‘envfit’ function in the vegan package. Spearman’s correlation test and the Mantel test were performed using the ‘ggcor’ package [49], to evaluate the relationships between soil factors and fungal communities. Random forest modelling [50] was applied to identify the main drivers of CMI by using the ‘randomForest’ [51] package, whereas the significance of the model and the predictor importance were determined using ‘therfutilities’ [52] and ‘rfpermute’ [53] packages. Linear regression was employed to assess the correlation between the CMI and its important predictors using the ‘basicTrendline’ [54] package.

## 3. Results

### 3.1. Soil Properties

Plantation management practices had a significant effect on all evaluated soil properties (Table 1). Compared with samples from the M0 and M2 plots, the pH was highest (*p* ≤ 0.05) in the soil from the M1 plots, in the following order: M1 > M0 > M2. The M0 soil had higher (*p* ≤ 0.05) SOC, NH_4_-N, and NO_3_-N contents than that from the other two sites, and the SOC and NH_4_-N in the M1 soil were significantly (*p* ≤ 0.05) higher than in the M2 soil. Compared with M0 and M1, the soil from the M2 plots had significantly (*p* ≤ 0.05) increased AP and AK, and there was a significant (*p* ≤ 0.05) difference in AK between the M0 and M1 soil.

### 3.2. SOC Fractions and CMI

Similar significant (*p* ≤ 0.05) changes were observed concerning C1, C2, C3, C4, CPI, and the CMI, under the different plantation management practices, in the following order M0 > M1 > M2 (Table 2). Compared with M0 and M1, M2 had significantly reduced L and LI (Table 2).

### 3.3. Shifts in Fungal Community Diversity and Composition

**Figure 1:**
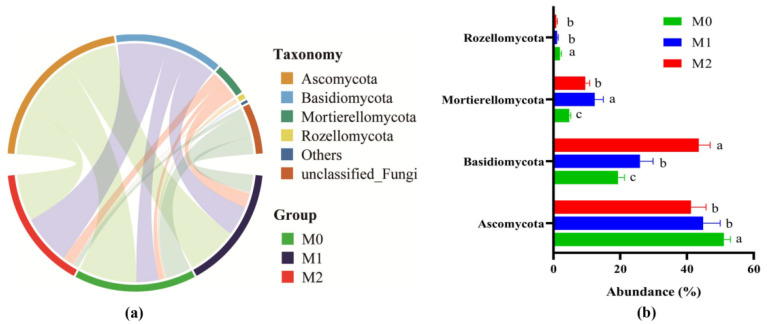
Characterization of the soil fungal community in soil samples from the three different management practices. (**a**) Venn diagrams of soil fungal OTUs; Fungal phyla with average relative abundance < 1% were merged and indicated as ‘Others’. (**b**) Comparative analysis for the composition of dominant fungal phyla. Different letters indicate significant variation among the treatments according to one-way ANOVA (LSD, *p* ≤ 0.05, *n* = 6).

In total, 2,135,898 fungal sequences were obtained from the 18 soil samples, after quality filtering, which was normalized to 30,807 sequences per sample. The fungal sequences were clustered into 932 OTUs, based on 97% sequence identity. Among these, 225 OTUs were common among the three treatments. The number of unique OTUs in M0, M1, and M2 was 148, 89, and 73, respectively (Figure S1). In all the soil samples, we identified four fungal phyla with relative abundances of over 1% (Figure 1), namely *Ascomycota* (45.73%), *Basidiomycota* (29.63%), *Mortierellomycota* (8.89%), and *Rozellomycota* (1.32%). We also identified six genera with relative abundances in excess of 1% (Table S1). These were *Saitozyma* (15.12%), *Mortierella* (8.83%), *Archaeorhizomyces* (3.93%), *Trichoderma* (2.30%), *Russula* (2.23%), *Metarhizium* (1.32%), and *Cladophialophora* (1.03%).

**Figure 2:**
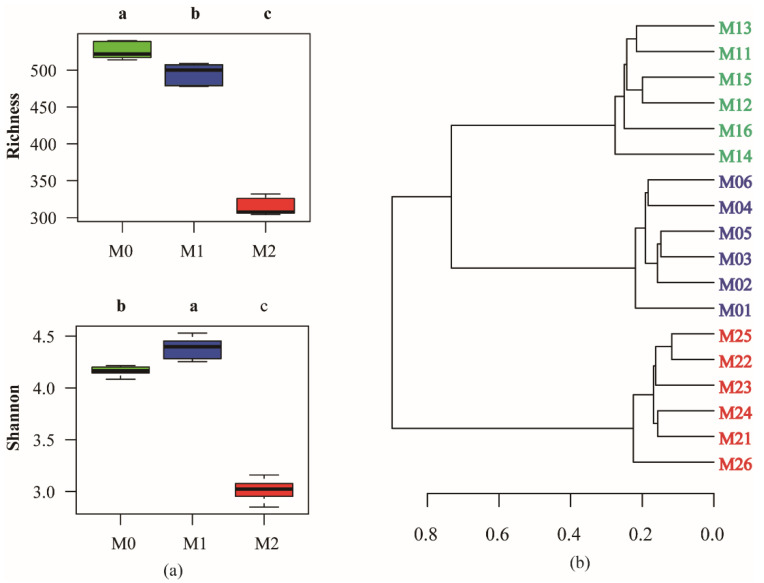
(**a**) Alpha-diversity indices of soil fungal community among three different management practices. Different letters indicate significant variation among the treatments according to one-way ANOVA (least significant difference [LSD], *p* ≤ 0.05, *n* = 6). (**b**) Hierarchical cluster diagram of soil fungal community. Blue, green, red numbers denotes the soil samples from undisturbed (M0), extensively managed (M1), and intensively managed (M2) bamboo plantations, respectively. Each group has six replicates.

We used two indices (the observed number of OTUs and the Shannon index) to assess the fungal alpha diversity (Figure 2a). A significant (*p* ≤ 0.05) difference was observed in the number of OTUs among the three groups in the following order: M0 > M1 > M2 (Figure 2a). Compared to M0, the Shannon index increased (*p* ≤ 0.05) in M1 and decreased (*p* ≤ 0.05) in M2 (Figure 2a). A hierarchical cluster analysis, based on Bray–Curtis distances, showed that the soil samples from the M0, M1, and M2 sites were clustered and differentiated into three significant groups (Figure 2b). The ANOSIM test also revealed significant (*p* ≤ 0.05) differences between M0, M1, and M2.

A comparison of the dominant phyla and genera is presented in Figure 1b and Table S1. M1 and M2 had reduced (*p* ≤ 0.05) the relative abundances of *Ascomycota*, *Rozellomycota*, Archaeorhizomyces, and Russula, relative to M0. The M1 and M2 soil had a higher (*p* ≤ 0.05) relative abundance of *Basidiomycota*, *Mortierellomycota*, *Trichoderma*, and *Mortierella* than the M0 soil. Compared to M0 and M1, *Metarhizium* was significantly (*p* ≤ 0.05) lower and *Saitozyma* was significantly (*p* ≤ 0.05) higher in soil from the M2 plots (Table S1).

### 3.4. Correlations between Soil Environmental Factors and Fungal Communities

**Figure 3:**
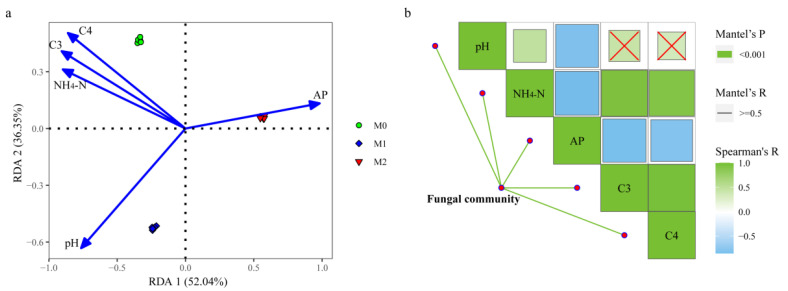
Redundancy analysis of fungal community structure and soil properties (**a**). AP—available P; C3—less labile fraction of oxidizable carbon; C4—nonlabile fraction of oxidizable carbon. Spearman’s correlation analysis and Mantel tests for fungal communities (**b**). Red crosses indicate *p* > 0.05. Edge width corresponds to the Mantel’s R value and the edge color denotes the statistical significance.

The outcomes of the RDA showed that the first two components could explain 88.39% of the total variation (Figure 3a). The soil pH (*r^2^* = 0.973, *p* = 0.001), NH_4_-N (*r^2^* = 0.902, *p* = 0.001), AP (*r^2^* = 0.979, *p* = 0.001), C3 (*r^2^* = 0.987, *p* = 0.001), and C4 (*r^2^* = 0.994, *p* = 0.001) significantly affected the fungal community structure. Furthermore, the Mantel test (Figure 3b) suggested a high correlation between the soil properties and the fungal communities, based on the pH (*r* = 0.640, *p* < 0.001), NH_4_-N (*r* = 0.744, *p* < 0.001), AP (*r* = 707, *p* < 0.001), C3 (*r* = 0.791, *p* < 0.001), and C4 (*r* = 0.697, *p* < 0.001).

### 3.5. Network Analysis of Soil Fungal Communities

To characterize the fungal–fungal interactions in bamboo soils, we structured a soil fungal network–consisting of 99 nodes and 1467 edges–based on correlations between the top 100 OTUs (Figure S2). The average degree (AD) and modularity (MD) were 29.636 and 1.049, respectively. Nodes were mainly assigned to four fungal phyla, including *Ascomycota* (58.59%), *Basidiomycota* (20.20%), *Mortierellomycota* (7.07%), and *Rozellomycota* (1.01%). Five OTUs were identified as keystone taxa and assigned to the following four genera: *Pyrenochaeta*, *Mortierella*, *Saitozyma*, and *Cladophialophora* (Table S2).

**Figure 4:**
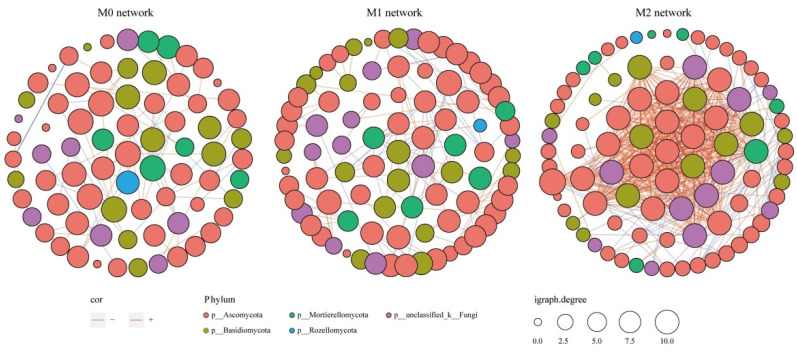
Co-occurrence networks of fungi in the bamboo soils from three management practices, based on Spearman’s correlation analysis between OTUs. Red (+) and blue (−) lines denote significant (ρ > |0.6|, *p* < 0.05) positive and negative correlations, respectively. The size of each node represents the connection number (degree). Nodes are colored by phylum.

To further investigate the fungal co-occurrence patterns within each soil preparation, three networks were constructed based on the OTU level (Figure 4). The network properties of the soil fungal communities are summarized in Table 3. The co-occurrence network analysis showed that the number of nodes and edges in the M2 and M1 network were higher than in the M0 networks; M1 and M2 networks had the same number of nodes, but M2 had an increased number of edges, compared to the M1. These indicated that the M2 network was comparatively more complex, relative to the M0 and M1 networks. Additionally, the M2 network had a higher average degree and average clustering coefficient than the M0 and M1 networks (Table 3). The number of negative links in the M0, M1, and M2 networks were 63, 111, and 74, respectively, accounting for 40.65%, 46.06%, and 12.03%, respectively, of the total number of corresponding links (Figure 4, Table 3).

### 3.6. Reliable Predictors of the CMI

**Figure 5:**
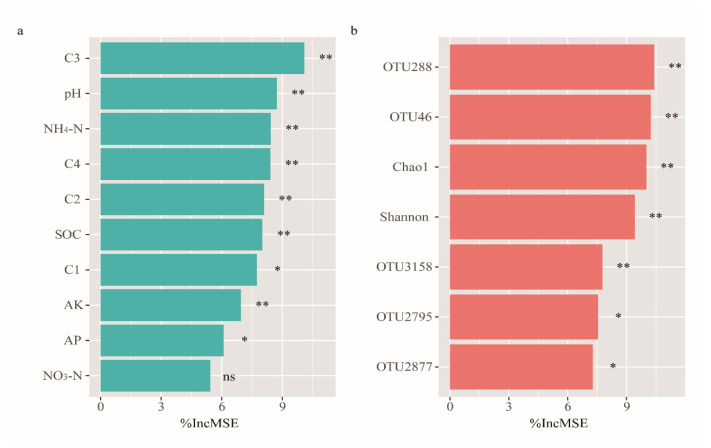
Random forest analysis showing the relative contribution of (**a**) Soil properties and (**b**) fungal community in determining the CMI. The fungal community data represent alpha indices (Shannon and Chao1) and relative abundance of keystone taxa (for each OTU in Table S2). ** *p* ≤ 0.01; * *p* ≤ 0.05; ns *p* > 0.05. IncMSE—the increase in mean square error.

The CMI predictions, made via random forest modeling, revealed that soil C3, pH, NH_4_-N, C4, C2, SOC, C1, AK, and AP were significantly responsible for the soil CMI (Figure 5a). C3 had the highest mean predictor importance (MPI) (10.08%), while 98.13% of variation was explained through modeling (*R^2^* = 0.981, *p* = 0.001), as seen in Figure 5a. Random forest models were also employed to determine the Shannon, Chao1, and keystone indices of the fungal communities responsible for the CMI (Figure 5b). OTU288 (*Mortierella*) had the highest MPI (10.40%), and 95.91% of variation was explained by modeling (*R^2^* = 0.957, *p* = 0.001), as shown in Figure 5b. The linear model showed that the CMI increased significantly with increasing C3 (*R^2^* = 0.997, *p* < 0.0001) and decreasing OTU288 (*Mortierella*) (*R^2^* = 0.784, *p* < 0.0001: Figure S3).

## 4. Discussion

### 4.1. Effects of Plantation Management Practices on Soil Properties and SOC Fractions

This study showed that SOC, C1, C2, C3, and C4 were reduced in M2 soil, compared with that from the M0 and M1 plots. Fontaine et al. [55] suggested that plant litter and root exudates are the principal sources of soil C. Another study showed that increasing plant diversity could significantly increase the amount of root exudates [56]. We also found that M1 significantly increased the soil pH, compared with M0. Girkin et al. [57] suggested that high concentrations of root exudates could cause a greater reduction in soil pH. Our previous study showed that bamboo plantations subjected to M0 experienced less disturbance than those subjected to M1 and M2, resulting in increased plant diversity and plant residue input [10]. Thus, changes in understory vegetation and litter inputs could explain the changes in the soil pH, as well as in the SOC and its fractions. Moreover, M2 soil had the lowest pH, mainly because of the use of N fertilizers, which are the primary drivers of soil acidification [58,59]. The soil from the M1 and M2 plots had lower NH_4_-N and NO_3_-N concentrations than the M0 soil. This may be because NH_4_-N and NO_3_-N are easily absorbed by plants [60] and plantation management increases the N uptake of Moso bamboo [61]. In contrast, the AP and AK contents increased significantly under M2, which was partially associated with the use of P and K fertilizers in these plantations.

### 4.2. Effects of Management Practices on Soil Fungal Community

Soil microbial diversity had a strong link to soil quality and the nutrient cycling rate [62]. Owing to their rapid response to soil changes, soil pH and nutrient levels are widely used to assess soil quality and microbial communities [63,64]. In our study, the fungal richness and diversity were lowest in the M2 soil, which may be due to the decreased soil physicochemical properties, compared with that of soil from the M0 and M1 plots. Soil microbial communities play a key role in soil function and ecosystem sustainability [65,66]. Chaer et al. [67] suggested that more abundant biodiversity indicates greater soil stability and Maček et al. [68] showed that lower fungal biodiversity causes unsustainable crop production and ecosystem instability. These studies suggested that M2 alters the stability of bamboo plantations and threatens ecosystem function. We also found that the selected soil physicochemical properties significantly influenced the soil fungal communities and, thus, were able to infer that changes in the soil properties caused by different bamboo management approaches, would contribute to variations in fungal communities. Soil pH is a key factor in shaping the soil fungal community because it may modify nutrient availability or limit fungal growth, affecting the diversity and composition of the fungal community [69,70]. In this study, the Shannon index was highest for M1 soil samples and lowest for M2 soil samples, which was consistent with their pH order, indicating that soil pH was the most influential factor in the Shannon index.

Our results demonstrated that *Ascomycota* and *Basidiomycota* were the dominant phyla in bamboo soils, which is consistent with the results of our previous study [71]. Numerous studies have reported that members of *Ascomycota* and *Basidiomycota* are key fungal decomposers in soil ecosystems [72,73]. The *Basidiomycetes* species can degrade lignocellulosic organic matter more effectively than other fungal groups [74,75], whereas *Ascomycetes* taxa have a limited ability to degrade litter containing recalcitrant lignin [76]. These findings indicated the ubiquity and importance of *Ascomycota* and *Basidiomycetes* in bamboo ecosystems, especially concerning the C cycle. Our results also indicated that M1- and M2-managed soils had a lower abundance of *Ascomycota* and *Rozellomycota* and a higher abundance of *Basidiomycota* and *Mortierellomycota*, compared to the M0 soil. Wu et al. [77] revealed that *Ascomycota* prefers to live in high-N soils, whereas *Basidiomycota* and *Mortierellomycota* are more suited to growth in low-N soils. According to Tian et al. [78], soil NO_3_-N content may affect the distribution of *Rozellomycota*. In our study, the shifts observed in these four fungal phyla were mainly due to changes in the soil N levels (NO_4_-N and NO_3_-N).

A network analysis identified *Pyrenochaeta*, *Mortierella*, *Saitozyma*, and *Cladophialophora* as keystone taxa. Previous studies have reported that the genus, *Pyrenochaeta*, is involved in the degradation of polysaccharides, such as cellulose, hemicellulose, pectin, and xylan [79,80]. Furthermore, Li et al. [81] confirmed that *Mortierella* species can compose recalcitrant substances and contribute to soil organic matter (SOM) storage. *Saitozyma* has the potential to decompose dead plant biomass because of its ability to incorporate C from cellulose [82]. Harsonowati et al. [83] found that *Cladophialophora* could promote nutrient uptake (N and K) from the soil and improve plant growth and fitness. Based on our results, keystones were the main contributors to variations in soil C content.

The results also indicated that M2 produced greater complexity of, and lower competition among the fungal network, compared with M0 and M1. Huang et al. [84] showed that the exogenous addition of glucose could decrease the complexity of fungal networks. Dundore-Arias et al. [85] found that an increased input of soil exogenous C could enhance microbial competition for C sources because of their niche overlap. The differences in plant residue input caused by the three plantation management approaches, evaluated herein, may partly contribute to shifts in the soil fungal network. Moreover, 87.97% of the fungal OTUs in the M2 soil were positive connections, revealing that the network was unstable [86]. This phenomenon may be due to the fungal OTUs producing co-fluctuations and positive feedback with environmental changes [87,88].

### 4.3. Effects of Plantation Management Practices on Soil CMI

The CMI reflects dynamic changes in soil C and has been used to assess the effect of plantation management practices on soil quality [89,90]. A higher CMI value suggests that the soil has higher potential to store C and reduce losses [90,91]. Moreover, higher CMI values reflect that soil C is being rehabilitated, whereas lower values indicate that the system is declining [35]. Our results indicated that M2 resulted in the lowest CMI among the three plantation management practices, indicating that soil managed in this manner had a low-quality C pool and could be at risk of degradation. A random forest analysis revealed that soil C3 and *Mortierella* were the best predictor variables of the CMI. Studies have indicated that C3 has stable molecular structures and changes slowly through microorganism activity, thereby playing an important role in SOC storage [18,92]. *Mortierella* is involved in the decomposition of plant litter and aromatic hydrocarbons [93,94] and Li et al. [81] elucidated that the *Mortierella* species can produce antibacterial compounds and chemically recalcitrant melanin to delay soil C turnover, contributing to SOC storage. Therefore, C3 and *Mortierella* can be used as indicators for evaluating SOC storage.

## 5. Conclusions

This study showed that Moso bamboo plantations subjected to M2 plantation management practices had lower SOC, oxidizable OC fractions, and CMI values, indicating that this approach is not beneficial for the accumulation and stability of SOC. The M1 approach increased soil pH, relative to M0 and M2. We also identified keystone fungal species in the studied bamboo soils, revealing complex interrelationships among the soil physicochemical properties, oxidizable OC fractions, and shifts in the fungal communities. Moreover, soil C3 and the relative abundance of *Mortierella* were the most important predictors of the CMI. These findings improve our understanding of the alteration of soil oxidizable OC fractions in Moso bamboo plantations, subjected to different management practices. In conclusion, fewer soil disturbances could increase the accumulation and stability of SOC and facilitate the sustainable development of bamboo ecosystems. These results can help us design suitable management practices to improve C sequestration and, thus, mitigate climate change in forest ecosystems.

## Figures and Tables

**Table 1:** Soil properties in bamboo soils from undisturbed (M0), extensively managed (M1), and intensively managed (M2) stands.

	M0	M1	M2
**pH**	4.93 ± 0.04 b	5.23 ± 0.06 a	4.69 ± 0.02 c
**SOC (g/kg)**	80.34 ± 1.63 a	47.18 ± 1.59 b	22.91 ± 0.59 c
**NH_4_-N (mg/kg)**	37.63 ± 1.77 a	31.12 ± 1.76 b	22.44 ± 2.93 c
**NO_3_-N (mg/kg)**	29.87 ± 4.12 a	12.28 ± 1.99 b	13.50 ± 1.54 b
**AP (mg/kg)**	35.49 ± 2.99 b	34.33 ± 1.14 b	71.88 ± 3.12 a
**AK (mg/kg)**	19.59 ± 1.75 b	14.16 ± 0.53 c	30.11 ± 1.02 a

SOC—soil organic carbon; AP—available P; AK—available K. Different lowercase letters within rows indicate significant difference at *p* ≤ 0.05 according to one-way ANOVA (LSD, *n* = 6).

**Table 2:** Soil oxidizable OC fractions and soil carbon management index in the treatments.

	M0	M1	M2
C1(mg/kg)	10.03 ± 0.65 a	6.53 ± 0.28 b	2.39 ± 0.17 c
C2 (mg/kg)	12.58 ± 0.77 a	7.43 ± 0.49 b	2.75 ± 0.18 c
C3 (mg/kg)	16.08 ± 0.77 a	10.11 ± 0.66 b	4.28 ± 0.20 c
C4 (mg/kg)	64.26 ± 1.73 a	37.07 ± 2.15 b	18.63 ± 0.54 c
CPI	1.00 ± 0.02 a	0.59 ± 0.02 b	0.29 ± 0.01 c
L	0.60 ± 0.03 a	0.65 ± 0.07 a	0.51 ± 0.02 b
LI	1.00 ± 0.06 a	1.08 ± 0.11 a	0.84 ± 0.04 b
CMI	100.00 ± 6.07 a	63.46 ± 4.91 b	23.93 ± 1.12 c

C1—very labile fraction of oxidizable carbon; C2—labile fraction of oxidizable carbon; C3—less labile fraction of oxidizable carbon; C4—nonlabile fraction of oxidizable carbon; CPI—Carbon Preference Index; L—Lability; LI—Lability Index; CMI—carbon management index. Different lowercase letters within rows indicate significant difference at *p* ≤ 0.05 according to one-way ANOVA (LSD, *n* = 6).

**Table 3:** Fungal network properties at different plantation management practices.

	M0	M1	M2
Node	69	84	85
Edge	155	241	615
Average degree	4.49	5.74	14.47
Average path length	4.05	3.73	3.19
Clustering coefficient	0.50	0.53	0.95

## Data Availability

The data that supports the findings of this study are available from the corresponding author upon reasonable request.

## Supplementary Materials

The following supporting information can be downloaded at: https://www.mdpi.com/article/10.3390/jof8060640/s1. Figure S1: Venn diagrams of soil fungal OTUs. M0, M1, and M2 indicated undisturbed, extensively managed, and intensively managed bamboo stands, respectively; Figure S2: Co-occurrence networks of fungal communities at OTU level colored by phylum in the Moso bamboo plantations under different management practices; Figure S3: Relationships between carbon management index and its important predictors. CMI, Carbon Management Index; C3, less labile fraction of oxidizable carbon; Table S1: Relative abundances of dominant genera in Moso bamboo soils under different management practices; Table S2: Keystones identified in the soil microbial communities.
