# Supplementary material for: Changes in Soil Organic Carbon Fractions and Fungal Communities, Subsequent to Different Management Practices in Moso Bamboo Plantations
Source: J Fungi (Basel). 2022 Jun 16;8(6):640. doi: 10.3390/jof8060640 (PMC9225535; doi:10.3390/jof8060640)
Supplement: Supplementary file 1 [file jof-08-00640-s001.zip › jof-1704382-supplementary.pdf]

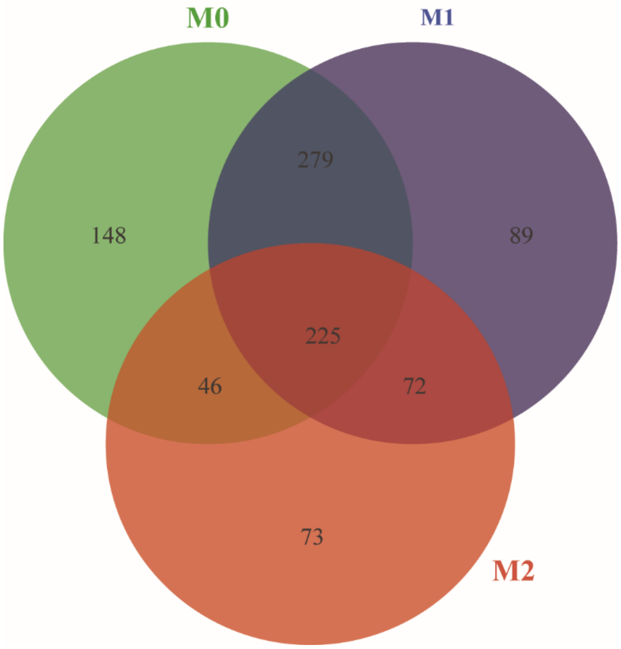

Figure S1. Venn diagrams of soil fungal OTUs. M0, M1, and M2 indicated undisturbed, extensively managed, and intensively managed bamboo stands, respectively

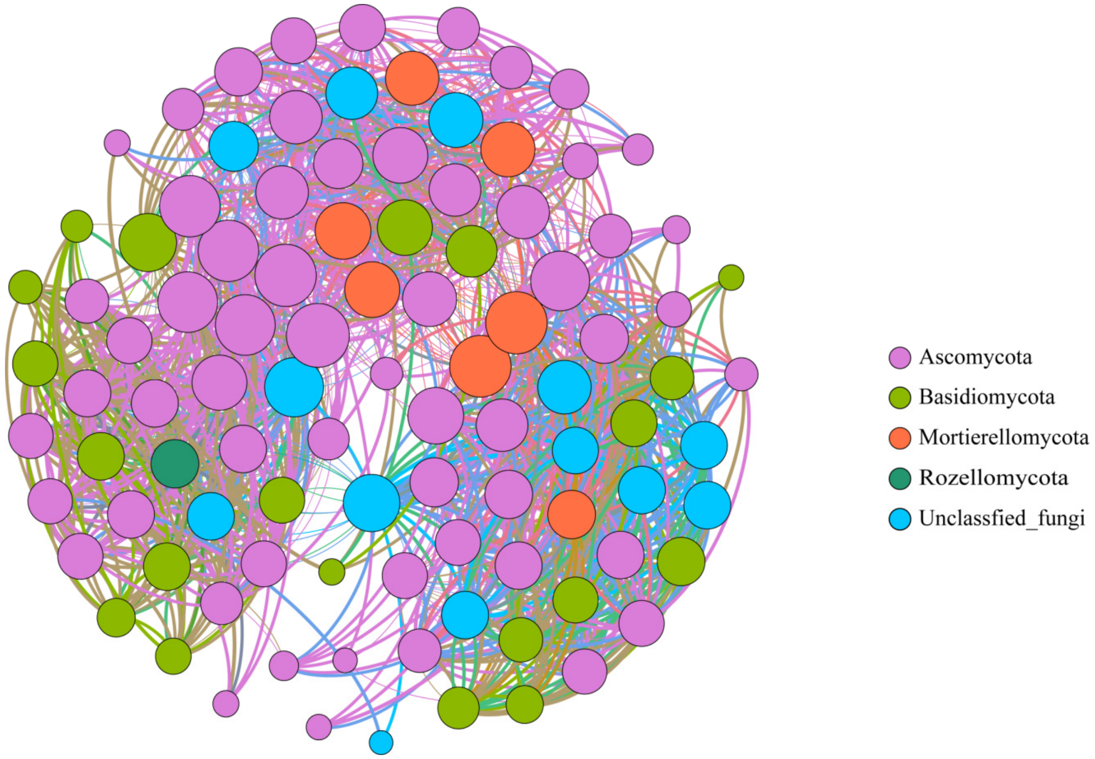

Figure S2. Co-occurrence networks of fungal communities at OTU level colored by phylum in the Moso bamboo plantations under different management practices.

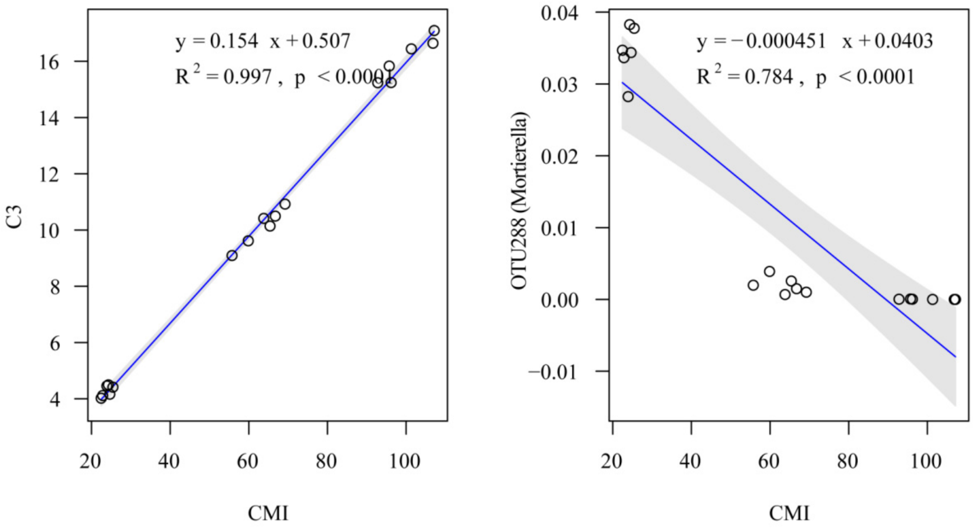

Figure S3. Relationships between carbon management index and its important predictors. CMI, Carbon Management Index; C3, less labile fraction of oxidizable carbon.

Table S1. Relative abundances of dominant genera in moso bamboo soils under different management practices.

Phylum	Genus	M0	M1	M2
Ascomycota	*Archaeorhizomyces*	6.10% ± 0.78% a	3.38% ± 0.63% b	2.31% ± 0.96% c
	Trichoderma	1.51% ± 0.28% c	2.27% ± 0.65% b	3.11% ± 0.68% a
	Metarhizium	1.42% ± 0.39% b	2.14% ± 0.53% a	0.42% ± 0.25% c
	Cladophialophora	0.77% ± 0.16% a	0.66% ± 0.16% a	1.66% ± 1.99% a
Basidiomycota	*Saitozyma*	3.47% ± 0.72% b	1.85% ± 0.51% b	40.04% ± 2.58% a
	Russula	6.69% ± 1.29% a	0.00% ± 0.01% b	0.00% ± 0.00% b
Mortierellomycota	*Mortierella*	4.75% ± 0.45% c	12.37% ± 2.54% a	9.36% ± 1.29% b

M0, M1, and M2 indicated undisturbed, extensively managed, and intensively managed bamboo stands, respectively. Different lowercase letters within rows indicate significant difference at $p \leqslant 0.05$ according to one-way ANOVA (LSD, n=6).

Table S2. Keystones identified in the soil microbial communities.

Node	Phylum	Class	Order	Family	Genus
OTU46	Ascomycota	Dothideomycetes	Pleosporales	Cucurbitariaceae	*Pyrenochaeta*
OTU288	Mortierellomycota	Mortierellomycetes	Mortierellales	Mortierellaceae	*Mortierella*
OTU2877	Basidiomycota	Tremellomycetes	Tremellales	Trimorphomycetaceae	*Saitozyma*
OTU2795	Ascomycota	Eurotiomycetes	Chaetothyriales	Herpotrichiellaceae	*Cladophialophora*
OTU3158	Mortierellomycota	Mortierellomycetes	Mortierellales	Mortierellaceae	*Mortierella*
